# Phenotypic and genotypic detection of antibiotic resistance of *Pseudomonas aeruginosa* isolated from urinary tract infections

**DOI:** 10.4314/ahs.v18i1.3

**Published:** 2018-03

**Authors:** Hisham A Abbas, Amira M El-Ganiny, Hend A Kamel

**Affiliations:** 1 Microbiology and Immunology Department, Faculty of Pharmacy, Zagazig University, Zagazig, Egypt; 2 Microbiology Department, Faculty of Pharmacy and Pharmaceutical Industries, Sinai University, Kantara, Egypt

**Keywords:** *Pseudomonas aeruginosa*, urinary tract infections, antibiotic resistance, resistance mechanisms

## Abstract

**Bakground:**

*Pseudomonas aeruginosa* is a major nosocomial uropathogen. It can tolerate a wide variety of physical conditions and many antibiotics by different resistance mechanisms.

**Objectives:**

This study aimed to investigate the mechanisms of antibiotics resistance in uropathogenic *P. aeruginosa* clinical isolates.

**Methods:**

Two hundred sixty six urine samples were collected from Zagazig University Hospitals, Zagazig, Egypt. *P. aeruginosa* isolates were identified using standard microbiological tests. The sensitivity to different antibiotics was determined by disc diffusion method. Anti-microbial resistance mechanisms were investigated using phenotypic methods and confirmed by PCR.

**Results:**

Fifty *P. aeruginosa* isolates were recovered. All isolates were MDR and were resistant to amoxicillin/clavulinic, sulphamethaxzole/trimethoprim, doxycycline and ceftazidime. Phenotypic detection of resistance mechanisms revealed that all strains have efflux mechanism, outer membrane porins, and AmpC β-lactamase; none of the strains showed ESBL activity and two of the imipenem resistant strains showed MβL activity. PCR analysis showed that all strains have MexAB-R, OprD and AmpC genes, 42 strains had PSE gene, while VEB and VIM genes were not detected.

**Conclusion:**

The resistance rates in *P. aeruginosa* were higher than global values; this resistance was attributed to several mechanisms. This high resistance is alarming and necessitates applying strict antibiotic prescription policies.

## Introduction

*Pseudomonas aeruginosa* is one of the most ecologically significant species among the genus *Pseudomonas. P. aeruginosa* is of extreme importance because of the widespread distribution of its strains in nature, its high intrinsic anti-bacterial resistance and its virulence^1^.

*P. aeruginosa* is an opportunistic, hospital-acquired pathogen that causes severe diseases in immuno-compromised individuals including urinary tract infection. Urinary tract infections (UTIs) are some of the most frequent bacterial infections, affecting 150 million people annually worldwide^2^. *P. aeruginosa* is the third most common pathogen associated with nosoomial catheter-associated UTIs^3^. Despite advances in anti-microbial therapy, the mortality and morbidity associated with *P. aeruginosa* induced UTIs remain significantly high^4^. One key reason for therapy failure is the increased level of antibiotic resistance among clinical *P. aeruginosa* isolates^5^. Thus, the detection of the underlying resistance mechanisms is critical for better management of this problem.

Many antibiotic resistance mechanisms have been reported in *P. aeruginosa* including: 1) Reduced expression or loss of OprD porin causing reduced antibiotic permeability^6^, 2) Over-expression of MexAB-OprM pump which increases antibiotic efflux^7^, 3) Production of β-lactams and aminoglycosides inactivating enzymes^8^, 4) Mutations of gyrases and topoisomerases which causes fluoroquinolone resistance^9^. These mehanisms in combination lead to multiple drug resistance^10^.

β-lactamases are hydrolytic enzymes that are responsible for the resistance to β-lactam antibiotics. β-lactamases have many types including extended spectrum β-lactamases (ESBLs), AmpC β-lactamases, carbenicillin hydrolysing β-lactamase, *Pseudomonas* specific enzyme (PSE) and metallo-β-lactamases (MβLs)^11^–^13^. ESBLs are encoded by different genes in *P. aeruginosa* including VEB gene^11^. MβLs are encoded by different genes including VIM (Verona Integron-encoded Metallo-β-lactamase) and IMP^14^.

Bacterial efflux pumps are greatly involved in the intrinsic resistance of Gram-negative bacteria. When overexpressed, efflux pumps can confer high resistance to previously effective antibiotics. Many efflux pumps transport a wide range of unrelated drugs and are known as multidrug resistance (MDR) efflux pumps. Four antibiotic efflux systems have been reported in *P. aeruginosa*. Mex-AB-OprM is the efflux system that is responsible for extrusion of β-lactams and quinolones^15^.

The outer membrane of *P. aeruginosa* represents a significant barrier that hinders the penetration of antibiotics. β-lactams and quinolones can only cross the outer membrane through the porin proteins. *P. aeruginosa* produces several porins such as OprD and OprF. Loss or diminished expression of OprD are frequently related to mipenem resistance i^16^.

This study was performed to detect antibiotic resistance profile of local *P. aeruginosa* isolated from urinary tract infections and to determine the underlying resistance mechanisms by phenotypic and genotypic methods.

## Material and methods

### Media and chemicals

Mueller Hinton broth, Mueller Hinton agar and antibiotic discs were purchased from Oxoid (Hampshire, UK). Ethidium bromide was obtained from Merck, Hohenburnn, Germany. MyTaqTM master mix was the product of Bioline Reagents Limited, UK, while Gene-Ruler 100 bp DNA Ladder was purchased from Thermoscientific Inc, USA. Other chemicals were of pharmaceutical grade.

### Bacterial strains

A total of 266 urine samples were collected from patients with urinary tract infections admitted to Urology Department, Zagazig University hospitals, Zagazig, Egypt. Only one sample was collected per patient. Samples were collected from clean-catch midstream fresh urine in sterile plastic jars. Samples were immediately transported to the Microbiological laboratory at Faculty of Pharmacy, Zagazig University, where they were immediately processed. *P. aeruginosa* identification was based on standard microbiological technique including: Gram staining, colony morphology, motility, pigment production, oxidase reaction, growth at 42 °C, gelatin liquefaction test and sugar utilization tests^17^.

### Anti-microbial susceptibility testing:

Anti-microbial susceptibility testing was performed on Mueller Hinton agar (MHA) plates by disc diffusion method and interpreted according to Clinical Laboratory Standards Institute guidelines (CLSI)^18^. The tested classes of antibiotics were penicillins, carbapenems, cephalosporins, β-lactam/β-lactamase inhibitor combination, aminoglycosides, tetracyclines, chloramphenicol, sulphonamides and fluoro-quinolones. The antibiotic discs included amikacin (30 µg), gentamicin (10 µg), piperacillin/tazobactam (100/10 µg), imipenem (10 µg), ceftazidime (30 µg), cefoperazone (10 µg), cefotriaxone (30 µg), cefotaxime (30 µg), cefepime (30 µg), amoxicillin/clavulanic acid 2:1 (30 µg), ciprofloxacin (5 µg), levofloxacin (5 µg), chloramphenicol (30 µg), doxycycline (30 µg), pipracillin (100 µg) and sulphamethoxazole /trimethoprim (1.25/23.75 µg).

### Phenotypic detection of resistance mechanisms: Phenotypic detection of MβL activity:

The imipenem-resistant *P. aeruginosa* isolates were investigated for MβL production by imipenem-Ethylene diamine tetra acetic acid combined disc test (IMP-EDTA CDT) as described previously by Yong et al.^19^ Briefly, overnight culture of the test organism was prepared and its turbidity was adjusted to a 0.5 McFarland standard and surface inoculated on MHA plate (9 cm in diameter). Two imipenem discs (10 µg) were placed at a distance of 4–5 cm from each other on the plate, and amount of 10 µL of 0.5M EDTA solution was added to one of them. The inhibition zones of the imipenem and imipenem-EDTA discs were compared after 18 h of incubation at 35 °C. Isolates were considered as MβL positive if the zone diameter of imipenem-EDTA disc was larger by more than or equal to 7 mm.

### Phenotypic detection of AmpC β-lactamases production:

AmpC β-lactamase production was phenotypically detected on the isolates that were resistant to cefoxitin according to Vanwynsberghe et al.^20^ Briefly, ceftazidime (30 µg) and cefotaxime (30 µg) disks were each placed at a distance of 20 mm from cefoxitin (30 µg) disk on a 9 cm-diameter MHA plate inoculated with the test *P. aeruginosa* isolate. The production of AmpC enzyme was confirmed when the zones of inhibition produced by either of the cephalosporins (ceftazidime or cefotaxime) when used in conjunction with cefoxitin were ≥5 mm larger than cephalosporins inhibition zone alone.

### Phenotypic detection of extended spectrum β-lactamases (ESBL) production:

A lawn culture of the organisms was made on a 9 cm-diameter MHA plate, as recommended by CLSI^21^. A disc which contained ceftazidime-clavulanate (30/10 µg) and ceftazidime discs (30 µg) were placed at distance of 20 mm. After an overnight incubation at 37°C, an increase of 5 mm in zone of inhibition of the combination discs in comparison to the ceftazidime disc alone was considered to be ESBL producer.

### Phenotypic detection of efflux pumps by ethidium bromide cartwheel (EtBr-CW) method

The ability of efflux pumps to expel ethidium bromide was assessed according to EtBr-CW method^22^. Trypticase Soya Agar (TSA) plates containing EtBr ranging from 0 to 4 mg/L (these concentrations were determined according to the bacterial MICs of EtBr) were freshly prepared on the same day of the experiment and kept protected from light. Overnight cultures of the tested bacterial isolates were prepared and adjusted to a 0.5 McFarland turbidity standard. The 9 cm-diameter TSA plates were divided into ten to twelve sectors forming a cartwheel pattern. The adjusted bacterial cultures were swabbed on the Et-Br-TSA plates starting from the center of the plate to the margin. After incubation of the plates at 37 °C for 16 h, the plates were examined under UV transilluminator (Cole-parmer, Vemon Hills, USA), the minimum concentration of EtBr that produced fluorescence of the bacterial mass was recorded. The isolates were considered Et-Br-CW-negative if they showed emission of fluorescence at 0.5–1 mg/L EtBr, EtBr-CW intermediate (emitting fluorescence at 2 mg/L) or EtBrCW-positive (emitting fluorescence only at 3–4 mg/L).

### Phenotypic detection of outer membrane permeability (OMP)

Assessment of MDR bacteria for OMP was performed by determination of the minimum inhibitory concentration (MIC) values for selected antibiotics in the presence and absence of EDTA (pH=7.2); a permeabilizer which chelates divalent cations that stabilize molecular interactions in the OM causing disruption of OMP. To avoid the effect of EDTA on bacterial growth, it was used at a concentration of ¼ MIC. A four-fold reduction in the antibiotic MIC or more in the presence of EDTA indicates OM reduced permeability activity.

The MICs of antibiotics and EDTA were determined by broth micro-dilution according to CLSI guidelines^23^. Müller Hinton broth (MHB) was inoculated with colonies of *P. aeruginosa* and broth was incubated with shaking at 37°C until the turbidity became equivalent to 0.5 McFarland standard. Then the bacterial suspension was diluted 1:100 in MHB medium. Two-fold serial dilutions of each drug were prepared in 96 wells microtiter plates in a final volume of 100 µL per well. Each well was inoculated with 100 µL of the previously prepared bacterial suspension and incubated at 37°C for 18 -20 h. MIC was defined as the lowest concentration of drug at which there was no visible growth of the organism. The results were recorded and interpreted according to CLSI^23^.

### Genotypic detection of resistance mechanisms by PCR.

The gDNA was extracted by picking a colony from an agar plate using a sterile pipette tip and resuspending it into 20 µL of distilled water. The mixture was vortexed for 10 s then incubated at 98 °C for 5 min. The lysate was centrifuged and the resulting supernatant was collected, diluted with distilled water at a 1:3 dilution ratio, and subjected to PCR analysis. Each PCR mixture contained 10 µL of MyTaqTM master mix; 1.5 µL of forward primer, 1.5 µL of reverse primer, 2 µL of gDNA template and nuclease free water to 20 µL. The primers used in this study are listed in Table 1. The PCR was performed in Biometra T-personal thermocycler (Goettingen, Germany). The PCR products as well as Gene-Ruler 100 bp DNA Ladder were separated on 1% agarose gel, stained with 2 µL of EtBr, and visualized by UV transilluminator and photographed.

**Table 1:** Primer sequence and amplicon size

Target gene	Primer sequence	Product size (bp)	Reference
mexA-F	CTCGACCCGATCTACGTC	503	Al-Grawi *et al.* 24
mexA-R	GTCTTCACCTCGACACCC		
mexR-F	GAACTACCCCGTGAATCC	411	Al-Grawi *et al.* 24
mexR-R	CACTGGTCGAGGAGATGC		
mexB-F	TGTCGAAGTTTTTCATTGATAG		Al-Grawi *et al.* 24
mexB-R	AAGGTCAC GGTGATGGT	280	
OprD-F	GCTCGACCTCGAGGCAGGCCA	242	Rodríguez-Martínez *et al.*^25^
OprD-R	CCAGCGATTGGTCGGATGCCA		
AmpC-F	GCTCCACCAACGGCTTCCTGAGGATGGCGTAGGC	124	Fazeli *et al.* 26
AmpC-R			
PSE-F	AATGGCAATCAGCGCTTC	698	Neyestana *et al.*^27^
PSE-R	GCGCGACTGTGATGTATA		
VEB-F	CATTTCCCGATGCAAAGCGT	648	Qing *et al.* 28
VEB-R	CGAAGTTTCTTTGGACTCTG		
VIM-F	GATGGTGTTTGGTCGCATA	390	Poire *et al.* 29
VIM-R	CGAATGCGCAGCACCAG		

Detection of Mex-ABR genes was done according to Al-Grawi et al.^24^ The cycling conditions were initial heating at 94°C for 3 min, then 32 cycles of 94°C for 30 sec, 57°C for 45 sec and 72°C for 1min and final extension at 72°C for 7min. OprD gene detection was performed according to Rodríguez-Martínez et al.^25^ and included heating at 94 °C for 2 min, then 29 cycles of 94 °C for 20 sec, 51 °C for 30 sec and 70 °C for 30 sec and final extension at 70 °C for 7 min. AmpC gene detection was according to Fazeli et al.^26^ and included heating at 94 °C for 2 min, then 29 cycles of 94 °C for 20 sec, 52.3 °C for 30 sec and 70 °C for 15 sec and final heating at 70 °C for 7 min. PSE gene detection was following Neyestanaki et al.^27^, heating at 94 °C for 2 min, then 29 cycles of 94 °C for 20 sec, 48.8 °C for 30 sec and 70 °C for 45 sec and finally heating at 70°C for 7 min. VEB gene detection was according to Qing et al.^28^ and include heating at 94°C for 2 min, then 30 cycles of 94 °C for 20 sec, 52.4 °C for 30 sec and 70 °C for 45 sec and finally heating at 70 °C for 7 min. VIM gene detection included heating at 94 °C for 2 min, followed by 29 cycles of 94 °C for 20 sec, 51 °C for 30 sec and 70 °C for 30 sec and final heating at70°C for7 min^29^.

## Results

### Antimicrobial susceptibility

**Figure 1:**
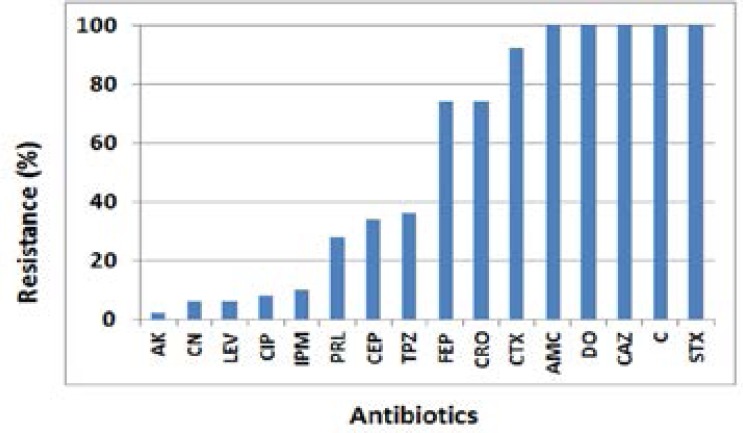
The antibiotic resistance of *P. aeruginosa* isolates

Only 50 *P. aeruginosa* strains were recovered from the 266 urine samples (18.7%). The resistance rates of *P. aeruginosa* isolates to the tested antibiotics were presented in Figure 1.

All the isolates were resistant to amoxicillin/clavulanic, sulphamethoxazole/ trimethoprim, doxycycline, chloramphenicol and ceftazidime. Resistance to cefotaxime was 92% and 74% for ceftriaxone and cefepime, respectively. The isolates showed low resistance to amikacin (2%), gentamicin (6%), levofloxacin (6%), ciprofloxacin (8%) and imipenem (10%). All of the *P. aeruginosa* isolates were multidrug resistant (MDR). The resistance pattern of the *P. aeruginosa* isolates was shown in Table 2.

**Table 2:** Antibiotic resistance pattern of the clinical *P. aeruginosa* isolates

Antibiotic resistance pattern	No of isolates
CN, AK, LEV, CIP, IMP, TPZ, CEP, PRL, FEP, CRO, CTX, DO, CAZ, C, STX, AMC	1
LEV, CIP, IMP, TPZ, CEP, PRL, FEP, CRO, CTX, DO, CAZ, C, STX, AMC	1
LEV, CIP, IMP, TPZ, CN, CEP, FEP, CRO, CTX, DO, CAZ, C, STX, AMC	1
TPZ, CEP, PRL, FEP, CRO, CTX, DO, CAZ, C, STX, AMC	11
TPZ, CN, CIP, FEP, CRO, CTX, DO, CAZ, C, STX, AMC	1
TPZ, CEP, FEP, CRO, CTX, DO, CAZ, C, STX, AMC	3
TPZ, PRL, FEP, CRO, CTX, DO, CAZ, C, STX, AMC	1
IMP, FEP, CRO, CTX, DO, CAZ, C, STX, AMC	1
FEP, CRO, CTX, DO, CAZ, C, STX, AMC	13
CRO, CTX, DO, CAZ, C, STX, AMC	4
IMP, CTX, DO, CAZ, C, STX, AMC	1
FEP, CTX, DO, CAZ, C, STX, AMC	2
CTX, DO, CAZ, C, STX, AMC	6
FEP, DO, CAZ, C, STX, AMC	2
DO, CAZ, C, STX, AMC	2

### Phenotypic detection of resistance mechanisms

**Figure 2:**
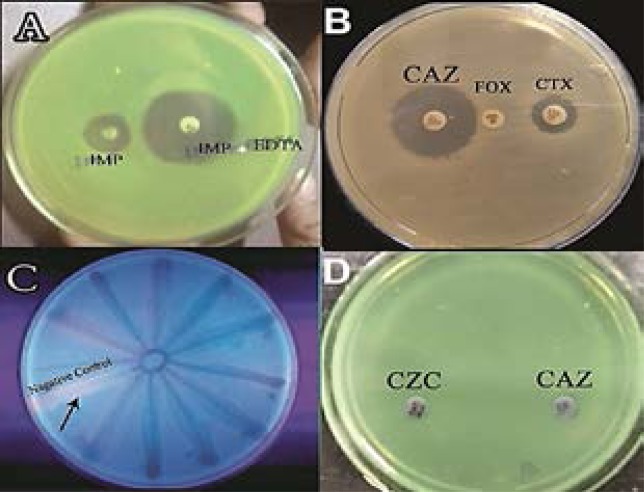
Phenotypic detection of resistance mechanisms. A, detection of MβL by IMPEDTA-CDT method, positive isolates showed ≥7 mm increase in zone of inhibition in presence of EDTA; B, detection of AmpC β- lactamase, positive isolates show that zones of inhibition produced by cephalosporins (CAZ or CXT) when used with cefoxitin (Fox) were 5 mm bigger than cephalosporins zone alone; C, detection of efflux pumps by EtBr-CW method, all the isolates did not show emission of fluorescence; D, detection of ESBL using ceftazidime (CAZ), and ceftazidime- clavulininc (CZC) discs, none of the isolates showed positive results.

The IMP-EDTA-CDT was performed for the 5 imipenem resistant isolates and the results revealed that 2 of these isolates were MβL producer (Fig. 2 A). All the 50 MDR *P. aeruginosa* isolates were phenotypically confirmed to produce AmpC β-lactamase (Fig. 2 B). All the MDR isolates were subjected to qualitative assessment for efflux pumps by EtBr-CW method, and all of the tested isolates showed efflux pump activity (Fig. 2 C). None of the MDR *P. aeruginosa* isolates were ESBL producers (Fig. 2 D). EDTA was used for phenotypic detection of OMP; generally EDTA potentiated the effect of different antimicrobial agents as it reduced the tested antibiotics MICs as indicated in Table 3.

**Table 3:** The MIC_50_ (µg/mL) of some antibiotics (without and with EDTA)

Antibiotic	MIC_50_	MIC_50_ in presence of EDTA
Amikacin	8	0.5
levofloxacin	2	0.125
cefotaxime	512	32
ceftazidime	1024	64

### Genotypic detection of resistance genes

**Figure 3:**
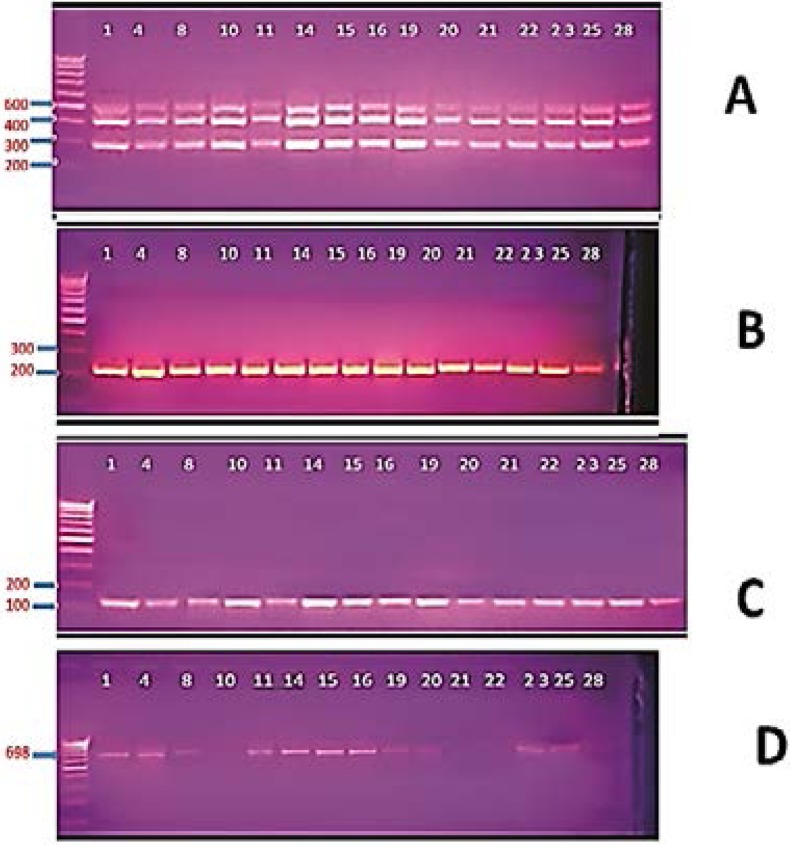
Gel electrophoresis of PCR products for detection of resistance genes genes in selected MDR isolates. A, detection of MexA-B-OprM genes, all the isolates gave triple bands of 280 bp, 411bp and 503bp representing MexB, MexR and MexA genes, respectively; B, detection of OprD gene, all isolates gave a single band at 242 bp; C, detection of AmpC gene, all the isolates gavea single band at 124 bp; D, detection of PSE gene, positive isolates gave a single band at 698 bp.

PCR was used to detect resistance genes. Multiplex PCR was used for amplification of MexA-B-R genes encoding efflux pumps, all the MDR isolates gave a triple band at 280, 411 and 503 bp matching the Mex-B-R-A genes (Fig. 3 A).

For OprD gene encoding outer membrane porins, all of the MDR isolates gave a single band at 242 bp matching OprD gene (Fig. 3 B). For amplification of AmpC gene encoding AmpC β-lactamase, all the MDR isolates gave a single band at 124 bp matching the AmpC gene (Fig. 3 C). In case of PSE gene, 42 of the MDR isolates gave a single band at 698 bp matching the PSE gene (Fig. 3 D). For VEB gene encoding ESBL enzyme and VIM gene encoding MβL, none of the tested isolates gave the 648 bp band matched to VEB gene or the 390 bp band matching VIB gene.

## Discussion

MDR isolates represents a prominent health problem in Egypt due to several factors including misuse of antibiotics and biocides^30^. In the current study, 50 *P. aeruginosa* isolates were recovered from urine samples, all the isolates were MDR (100%), this MDR rate was higher than rates reported previously in Egypt which ranged from 34% – 56%^31^–^33^; this may reflect higher resistance rate in case of urinary tract infections. Other global studies showed even lower MDR rates; 5.9% in Canada^34^, 19% in Germany^35^, 19.6 % in Malaysia^36^ and 20.7% in Nepal^37^. This MDR rate is alarming and necessities application of strict antibiotic prescription policies in our country.

The resistance rates were low to amikacin (2%), gentamicin (6%), levofloxacin (6%), ciprofloxacin (8%) and imipenem (10%). Previous studies also reported that amikacin and imipenem were the most effective drugs against *P. aeruginosa*^26^,^31^. Higher resistance rates were reported for pipracillin (28%), cefoperazone (34%), piperacillin/tazobactam (36%) cefotriaxone (74%), cefepime (74%), and cefotaxime (92%), while all the isolates were resistant to ceftazidime, chloramphenicol, doxycycline, amoxicillin/clavulanic and sulphamethoxazole/ trimethoprim. Selection of curative antibiotic should always depend on the results of antimicrobial susceptibility testing to avoid therapy failure. Inappropriate therapy has been associated with increased mortality in *P. aeruginosa* infections^38^.

In the current study, phenotypic detection of MβL production reveled that only 2 of the 5 imipenem resistant isolates (40%) were MβL-positive. A previous Egyptian study reported relatively similar results where 27% of *P. aeruginosa* were MβL producers^39^. PCR analysis showed that VIM gene (encoding MβL) was not detected in any of our isolates. High prevalence of VIM gene (58–61%) was reported previously in *P. aeruginosa*^40^, Probably the MβL activity in our study is attributed to other MβL genes or another mechanism. Several mechanisms of imipenem resistance have been reported previously including secretion of carbapenemases, increased expression of efflux systems, and reduced porin expression^41^. The isolation of carbapenem resistant strains is alarming and requires restriction on prescription of these valuable drugs

Phenotypic analysis showed that none of our isolates was an ESBL producer, this was comparable to the low occurrence (7.4%) reported previously in Egypt^40^. While Lin et al.^42^ found that 29% of his strains were ESBL producers. PCR was used for detection of VEB gene encoding ESBLs, the gene was not detected in any of the isolate which is compatible with our phenotypic results. Zafer et al.^40^ reported that 10.4% of ESBL-producing *P. aeruginosa* isolates were positive for VEB gene.

All of the tested isolates were AmpC β-lactamase producer. Chika et al.^43^ found that 36% of *P. aeruginosa* isolates were AmpC producers. The presence of AmpC -lactamase enzyme was confirmed by PCR, all of the *P. aeruginosa* isolates harbored the AmpC gene. Similarly, Fazeli et al.^26^ found that all the *P. aeruginosa* isolates had AmpC gene.

The *Pseudomonas* specific enzyme (PSE) belong to Class A carbenicillin hydrolysing β-lactamases that confer resistance to carbenicillin and pipracillin. The presence of PSE gene was tested by PCR, 42 isolate (84%) were found to have the PSE gene, it is worth mentioning that the 8 PSE-negative isolates were susceptible to pipracillin. Cho et al.^44^ reported lower rate where only two out of 61 *Pseudomonas* isolates (3.3%) harbored PSE gene. The AmpC and PSE are the main hydrolyzing enzymes that confer β-lactam resistance in our study.

The efflux system contributes to the natural bacterial resistance to a wide range of antibiotics and detergents^45^,^46^. In our study, active efflux was detected in all isolates. This was in accordance with Rana et al.^47^ who reported presence of active efflux in all MDR isolates. Presence of MexABR efflux system was confirmed by amplification of this operon using multiplex PCR. All of the *P. aeruginosa* isolates were positive to the three tested genes. This was in accordance with Al-Grawi et al.^24^ who found that all of the *P. aeruginosa* isolates were positive to the three genes.

Our study also investigated the outer membrane reduced permeability as a resistance mechanism; assay was performed with EDTA as a permeabilizer. The MIC values for tested antibiotics was determined in the presence and absence of sub MIC (1/4 MIC) of EDTA. Our results showed that EDTA has augmenting effect on different anti-microbial agents; EDTA reduced the MICs of various anti-microbials by up to 16 folds. This was comparable with the results of Ayres et al.^48^ who reported that EDTA was greatly enhanced the activity of various antibiotics and biocides against Gram negative isolates.

## Conclusion

This study revealed that the resistance rates of local *P. aeruginosa* were higher than worldwide values. This high resistance was attributed to several mechanisms including efflux pumps, reduced activity of outer membrane porins, production of AmpC and PSE β-lactamases. This high MDR resistance rate is alarming which necessitates applying strict antibiotic usage and prescription policies. Also selection of curative antibiotics should depend on the anti-microbial susceptibility results.
